# Effects of FTY720 (Fingolimod) on Proliferation, Differentiation, and Migration of Brain-Derived Neural Stem Cells

**DOI:** 10.1155/2016/9671732

**Published:** 2016-10-18

**Authors:** Botao Tan, Zeruxin Luo, Yan Yue, Yuan Liu, Li Pan, Lehua Yu, Ying Yin

**Affiliations:** ^1^Department of Rehabilitation Medicine, The 2nd Affiliated Hospital of Chongqing Medical University, Chongqing 400010, China; ^2^Physical Examination Center, Chongqing General Hospital, Chongqing, China; ^3^The 3rd Department of Research Institute of Surgery, Daping Hospital, The Third Military Medical University, State Key Laboratory of Trauma, Burns and Combined Injury, Chongqing, China; ^4^Kuanren Rehabilitation Hospital, The 2nd Affiliated Hospital of Chongqing Medical University, Chongqing, China

## Abstract

Insufficient proliferation, differentiation, and migration are the main pitfalls of neural stem cells (NSCs) in reparative therapeutics for the central nervous system (CNS) diseases. The potent lipid mediator sphingosine-1-phosphate (S1P) regulates cells' biological behavior broadly in the CNS. However, the effects of activating S1P on NSCs are not quite clear. In the current study, FTY720 (Fingolimod), an analog of S1P, was employed to induce the proliferation, differentiation, and migration of cultured brain-derived NSCs. The results indicated that proliferation and migration ability of NSCs were promoted by FTY720. Though we observed no obvious neuron prefers differentiation of NSCs, there were more protoplasmic astrocytes developed in the presence of certain concentration of FTY720. This work gives more comprehensive understanding of how FTY720 affects NSCs.

## 1. Introduction

Proliferation, differentiation, and migration properties of neural stem cells (NSCs) provide a promising future in repairing the injured or degenerated disorders of central nervous system [1]. However, the limitations of NSCs such as insufficient proliferation, failing of differentiating towards desired cell types, and not being able to migrate efficiently make it still a long way of utilizing them in clinical practice [2]. It was pointed out that biological behavior of NSCs was controlled by various intrinsic characteristics and extrinsic signals [3–5]. Among these, lipid mediators would be one of the undervalued candidates in regulating NSCs proliferation, migration, and differentiation.

Potent lipid mediator sphingosine-1-phosphate (S1P) as one of transduces intracellular signals plays a critical role in cells' biological behavior in the CNS by activating sphingosine-1-phosphate receptors (S1PRs) [6–8]. S1PRs participate in the anti-inflammatory process of microglia and proliferation of astrocytes, but their roles on NSCs have seldom been reported [9, 10]. Evidence showed that S1PRs are expressed in NSCs and the activation of S1PRs, especially S1P1, would regulate the migration and angiogenesis of NSCs [11, 12]. With the booming number of studies on stem cell transplantation or endogenous NSCs in CNS diseases, it is pending to demonstrate the role of S1PRs/S1P in regulating NSCs in detail.

In the present study, we employed a structural analog to S1P: FTY720, which is known as an oral drug for multiple sclerosis, to intervene in primary cultured NSCs [13]. We found out that migration and proliferation of NSCs were positively affected by FTY720. Though no significant differences of neuron preferring differentiation were found, there were more protoplasmic astrocytes developed in the presence of certain concentration of FTY720. This study gives more understanding of FTY720 in biological behavior of NSCs.

## 2. Materials and Methods

### 2.1. Culture of Neural Stem Cells

All experiments were approved by the Animal Care and Experimental Committee of Chongqing Medical University. NSCs were collected from embryonic 13.5 days (E13.5) of Sprague-Dawley rats as described previously [14, 15]. Briefly, the telencephalon was rapidly dissected and mechanically dissociated into single-cell suspension in tubes containing 0.25% trypsin. Single-cell suspension was then transferred to growth medium consisting of NB (Gibco, USA) + 2% B27 (Gibico, USA) supplemented with 20 ng/mL human recombinant basic fibroblast growth factor (bFGF; Invitrogen, USA) and 20 ng/mL epidermal growth factor (EGF; Invitrogen, USA). This also referred to basic condition medium. Then, cells were cultured at 37°C under a humidified atmosphere containing 5% CO_2_. Half of the medium was replaced every other day. Secondary or tertiary neurospheres were used for subsequent experiments.

For Brd-U incorporation, NSCs were incubated in growth medium containing 10 mM Brd-U (Sigma, USA) for 18 hours before immunocytochemical analysis.

### 2.2. FTY720 Intervention

To examine the effect of FTY720 (FTY720-P: FTY720 phosphate: Cayman, USA) [16], NSCs were divided into four groups according to the concentration of FTY-720 (0 nM, 1 nM, 10 nM, and 100 nM, diluted in DMSO) in basic culture medium within (for differentiation) or without (for CCK-8 testing and the sphere number counting) 10% fetal bovine serum (HyClone, Thermo, USA). 0 nM FTY720 was recognized as the control group. The differentiated cells were fixed and immunochemically stained with specific markers of neurons, astrocytes, and oligodendrocytes.

### 2.3. CCK-8 Assay

Cell counting kit-8 (CCK-8; Dojindo, Japan) was processed following the instructions, and passage cells of 100 *μ*L were plated onto 96-cell plates at the concentration of 1 × 10^5^ cells/mL and then cultured in 100 *μ*L basic culture medium. Twenty-four hours later, FTY720-P of different concentration (0 nM, 1 nM, 10 nM, and 100 nM) was added separately. 10 *μ*L CCK-8 solution was added 48 hours later and then measured the absorbance at 450 nm. The fold change of proliferation rates was calculated based on the absorbance results and the rate in control group normalized to 1. As for proliferated neural stem sphere counting, FTY720-P was added into the medium and the number of stem spheres was calculated 48 hours later.

### 2.4. Identification of NSCs and Differentiated Neural Cells

After 30 min fixation in 4% paraformaldehyde, cells were washed three times with 0.01 M PBS and incubated in 0.5% Triton-100. 1% BSA was used prior to an overnight incubation with the primary antibodies. Anti-Brd-U (1 : 800, Sigma, USA), anti-Ki67 (1 : 200, Cell Signaling), and anti-Nestin (1 : 400, Millipore, USA) were used as stem cell markers. Anti-*β*-tubulin III (1 : 800, Sigma, USA), anti-GFAP (1 : 160, Sigma, USA), anti-A2B5 (1 : 200, Abcam, USA), anti-Olig2 (1 : 500, Abcam, USA), and anti-CC1 (1 : 200, Life Science Research, USA) were used as specific cell type makers. Anti-S1P1 (1 : 100, R&D System, USA) or anti-S1P2 (1 : 200, Novus Biologicals, USA) were used to confirm the expression of S1P receptors in NSCs. FITC-conjugated goat secondary antibodies (1 : 100, Zhongshan, China) and TRITC-conjugated goat secondary antibodies (1 : 100, Zhongshan, China) were used for labeling the different source of primary antibodies separately. Subsequently, the samples were stained with DAPI nuclei staining prior to confocal laser scanning microscopy (Leica, SP2, Germany).

### 2.5. Transwell (Boyden Chamber) Cell Migration Assay

In a total volume of 600 *μ*L basic culture medium containing FTY720-P was added at the 24-cell plates coated with poly-L-lysine (Sigma, USA) while 100 *μ*L neurospheres cells were plated inside the Boyden Chamber at the density of 1 × 10^6^ cells/mL [17]. Eighteen hours later, the chambers were taken away, and the cells in each lower chamber were stained with crystal violet for the number analyzed.

### 2.6. Western Blot Analysis

The differentiated cells or NSCs were lysed (N-PER, Thermo Fisher, USA) and centrifuged at 14,0006 ×g for 20 minutes. Protein concentration of the supernatant was determined using BCA protein assay kit (Beyotime Biotechnology, China). An equivalent amount of protein samples was loaded on SDS-polyacrylamide gels and transferred to PVDF membranes (Millipore, USA). The blots were blocked and subsequently probed with anti-GFAP (1 : 400, Sigma, USA), anti-*β*-tubulin III (1 : 1500, Sigma, USA), anti-F-actin (Wanlei Biotechnology, China), and anti-Olig2 (1 : 2000; Abcam) overnight. Secondary horseradish peroxidase-conjugated antibodies (1 : 5000; Zhongshan Biotechnology, China) were used and finally, blots were detected with enhanced chemiluminescence (ECL) detection reagents (Thermo Fisher, USA). *β*-Actin was recognized as the internal control in a parallel running. Bio-Rad Image Lab Software Version 5.2.1 or Image J Version 1.50i was used for optical densities quantification as we did previously [18]. Separate experiments were conducted for three times.

### 2.7. Statistical Analysis

The number of neurospheres was counted and averaged in a total of 5 systematic fields (same location of the 24-well plates in each group) under a 10x objective, and this was repeated five times independently. The percentage of differentiated cells were calculated as *β*-tubulin III, GFAP, CC1, or Olig2 positive, while GFAP positive astrocytes have two types based on their morphology, all divided by the total number of differentiated cells (DAPI-labeled) at 5 random fields under a 40x objective for each group in five independent experiments. Data were presented as mean ± SD. Statistical analysis was performed using one-way analysis of variance (ANOVA) followed by Dunnett's multiple comparison tests. *P* < 0.05 was considered to be statistically significant.

## 3. Results

### 3.1. Primary Cultured NSCs Have the Ability to Differentiate and Proliferate

**Figure 1:**
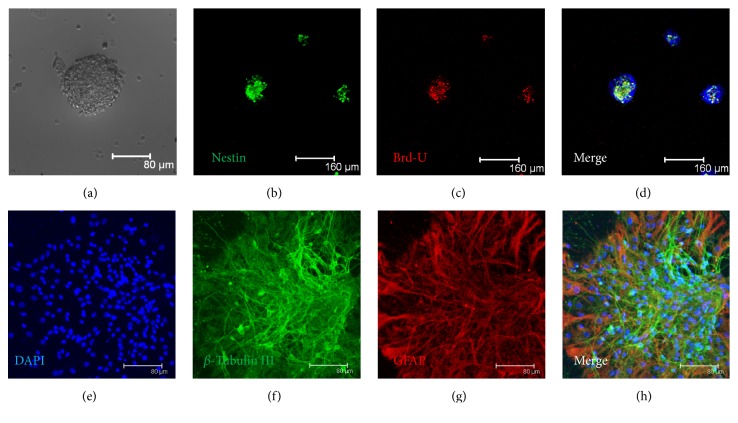
Identification of primary cultured NSCs* in vitro*. (a) Representative neurospheres in culture. ((b)–(d)) Immunocytochemical staining of purified NSCs with Nestin ((b), green) and Brd-U ((c), red) and merged with nucleus staining DAPI ((d), blue) (bar = 160 *μ*m). ((e)–(h)) Nucleus staining ((e), blue) and immunocytochemical of differentiated astrocytes with *β*-tubulin III ((f), green) and GFAP ((g), red) and merged with (h) (bar = 80 *μ*m).

Proliferating NSCs formed neurospheres in suspension on the following days, and they are Nestin (neural stem cell specific marker) positive (Figures 1(a) and 1(b)). Detection of DNA replication by Brd-U immunostaining of the NSCs spheres confirmed that these cells undergo proliferation (Figure 1(c)). After withdrawal of bFGF and EGF and fetal bovine serum addition, the spheres differentiated into neurons and astrocytes as identified by staining of neuron-specific marker *β*-tubulin III and astrocyte-specific marker GFAP, which testified that the NSCs have multiple differentiation potentials (Figures 1(g) and 1(h)). Besides, the neurospheres express S1P1 as it is double positive with Nestin (see Supplement Figure 1 in Supplementary Material available online at http://dx.doi.org/10.1155/2016/9671732).

### 3.2. FTY720 Promotes Proliferation of NSCs

**Figure 2:**
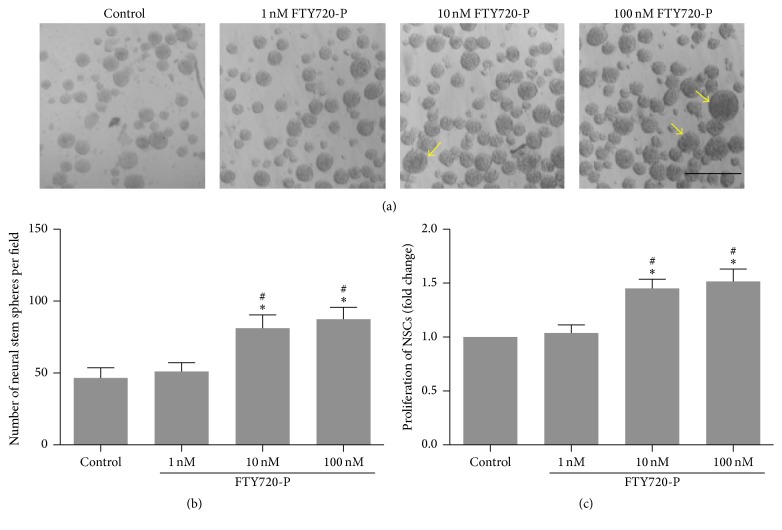
The proliferation of NSCs in the presence of FTY720. ((a)-(b)) Representative images of neurospheres and number quantification in response to FTY720. Bar = 500 *μ*m. Arrows indicated lagers sizes of the neurospheres. (c) Proliferation rate indexes in the presence of FTY720. Fold changes in proliferation were calculated by the absorbance of 450 nm of CCK-8 assay. Data represent the mean ± SD; *n* = 5 in each group; ^*∗*^ *P* < 0.05 compared with control group; ^#^ *P* < 0.05 compared with 1 nM FTY720 group, by ANOVA followed by Dunnett's multiple comparison tests.

There was an obvious increase in numbers of NSCs 48 hours after FTY720 intervention. What is more, some of the spheres were bigger in the 10 and 100 nM FTY720 groups compared to control and the 1 nM FTY720 groups (Figures 2(a) and 2(b); Supplement Figure 2). The result was further confirmed by CCK-8 assay (Figure 2(c)). NSCs in the higher concentration groups had stronger proliferation ability, which is 1.45- and 1.52-fold increase compared to the control group, *P* < 0.05. However, it seemed that FTY720 did not affect the proliferation rate of neural stem spheres at the concentration of 1 nM.

### 3.3. FTY720 Induces Differentiation of NSCs to Protoplasmic Astrocytes

The differentiating cells began to migrate out from the spheres 2~3 days later and developed morphological characteristics of neurons with spherical cell bodies and neurite-like processes or glial cells, including larger protoplasmic and slim fibrous outline astrocytes or short processes oligodendrocytes.

**Figure 3:**
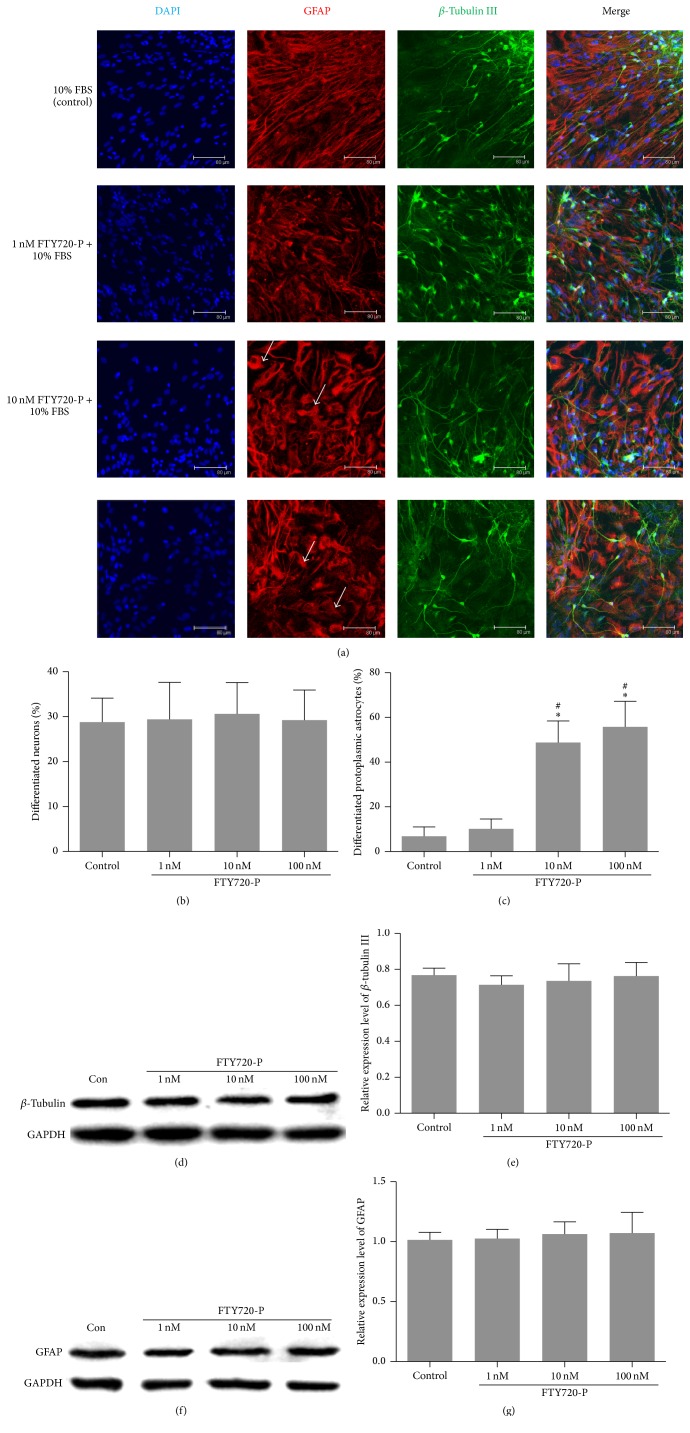
Differentiation of NSCs into neurons and astrocytes in the presence of FTY720. (a) Representative images of neurons (*β*-tubulin III positive, in green) and astrocytes (GFAP positive, in red) in different groups. Bar = 80 *μ*m. Arrows indicate protoplasmic astrocytes. ((b)-(c)) Quantification of the proportion of differentiated neurons and astrocytes. *n* = 5. ((d)-(e)) Western blot analysis of *β*-tubulin III protein in different groups. *n* = 3 in each group. ((f)-(g)) Western blot analysis of GFAP protein in different groups. *n* = 3 in each group. ^*∗*^ *P* < 0.05 compared with control group; ^#^ *P* < 0.05 compared with 1 nM FTY720 group, by ANOVA followed by Dunnett's multiple comparison tests.

Differentiation of neurons and astrocytes was confirmed by immunocytochemical staining with specific marker *β*-tubulin III and GFAP (Figure 3(a)). Quantification analysis showed no neuron prefers differentiation of the NSCs because there were no significant differences in the number of neurons among groups, with 28.8 ± 5.3%, 29.4 ± 8.2%, 30.6 ± 6.9%, and 29.2 ± 6.7% separately, *P* > 0.05 (Figures 3(a) and 3(b)). Western blot analysis showed similar results (Figures 3(d) and 3(e)).

However, the proportion of protoplasmic astrocytes obviously differs among groups. Larger cell body protoplasmic astrocytes (also known as type 1 astrocytes) were frequently observed in higher dose (10 nM and 100 nM) FTY720 groups, at 48.8 ± 8.8% and 55.8 ± 11.4%, respectively, which increased significantly versus control and 1 nM FTY720 group, with 6.8 ± 4.3% and 10.2 ± 4.3% separately, *P* > 0.05 (Figures 3(a) and 3(c)). Meanwhile, the smaller fibrous astrocytes (also known as type 2 astrocytes) were more likely to be seen in the control and 1 nM FTY720 group (data not shown). To determine whether it is the total number or only the relative frequency of protoplasmic astrocytes that increased, we next tested the protein level of GFAP using western blot. No differences of the relative GFAP protein level among groups were found, *P* > 0.05 (Figures 3(f) and 3(g)).

**Figure 4:**
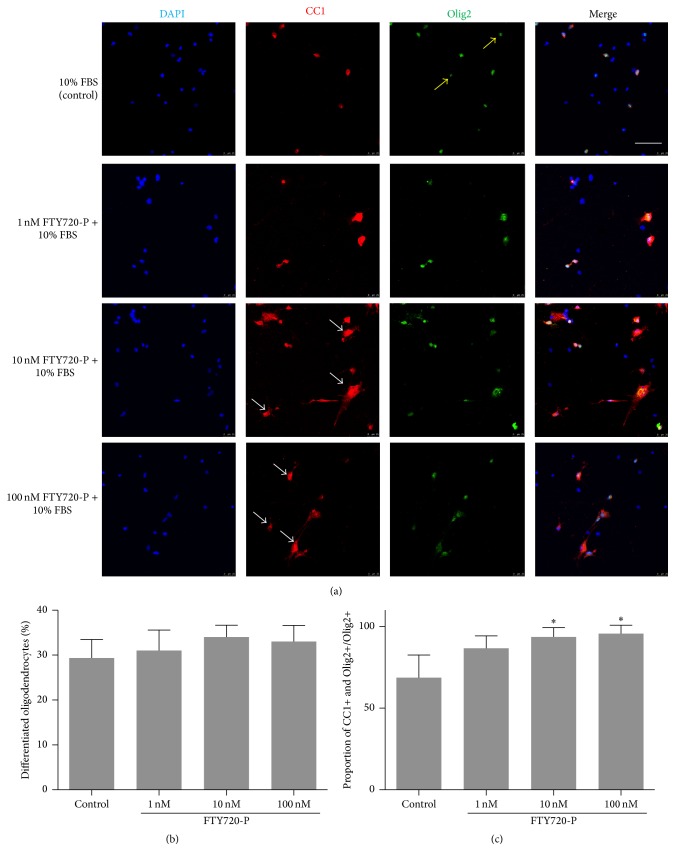
Differentiation of NSCs into oligodendrocytes in the presence of FTY720. (a) Representative images of differentiated oligodendrocytes in different groups. Matured oligodendrocytes are both Olig2 (Green) and CC1 (Red) positive. Parts of the Olig2+ cells in the control group did not express CC1 (yellow arrows) while more morphological matured (white arrows) oligodendrocytes were seen in higher FTY720 concentration groups. Bar = 50 *μ*m. ((b)-(c)) Quantification of the proportion of differentiated oligodendrocytes. *n* = 3 in each group. ^*∗*^ *P* < 0.05, compared with control group, by ANOVA, followed by Dunnett's multiple comparison tests.

To explore the differentiation of oligodendrocyte lineage, we stained the cells with Olig2 and CC1, and both were oligodendrocyte makers. There was a trend of increasing proportion of olig2 positive oligodendrocytes in the higher FTY720 groups, but lacking of significant differences, *P* > 0.05 (Figures 4(a) and 4(b)). Nevertheless, more morphological matured oligodendrocytes were seen in higher concentration groups, and the proportion of CC1/Olig2 double positive cells was more in FTY groups (Figure 4(c)).

### 3.4. FTY720 Promotes Migration of NSCs

**Figure 5:**
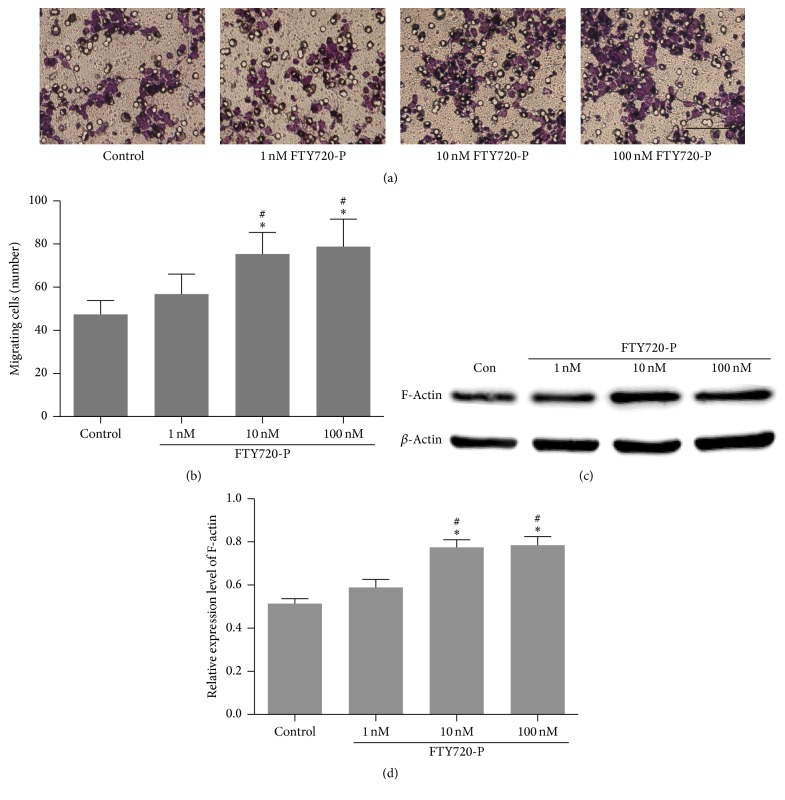
Quantification analysis of migrated cell numbers with FTY720 intervention. ((a)-(b)) Number of migrated cells from upper Boyden Chamber with crystal violet staining (bar = 100 *μ*m). Migrated cells were counted by crystal violet staining and averaged three random microscope fields of each sample; five independent experiments were done. ((c)-(d)) Relative expression level of F-actin among groups. Mean ± SD and *n* = 5 in each group. ^*∗*^ *P* < 0.05 compared with control group; ^#^ *P* < 0.05 compared with 1 nM FTY720 group, by ANOVA followed by Dunnett's multiple comparison tests.

To assess the effects of FY720 on NSCs migration, Boyden Chamber and crystal violet staining were used in the experiment. The results showed promoted NSCs migration in a concentration-dependent manner 18 hours following FTY720 intervention. With the increase of FTY720 concentration, the number of cells migrating to the lower membrane of the chamber grows (Figure 5(a)). Significantly higher number of migrating cells were found in the 10 nM and 100 nM FTY720 groups (75.4 ± 10.0 and 78.8 ± 12.7, resp.) in comparison with control (47.6 ± 6.4, *P* = 0.001). The 1 nM FTY720 group showed a little higher but no significant increase in the number of migrating cells (56.8 ± 9.2, *P* > 0.05 compared with control group) (Figure 5(b)).

Previous studies reported that filamentous actin (F-actin) polymerization is required for ligand-induced cell chemotaxis [19]. We then examined the protein expression level of F-actin in the NSCs. Consistent with the result in Boyden Chamber assay, F-actin was evidently upregulated in NSCs stimulated with 10 nM and 100 nM FTY720, *P* < 0.05, compared with control group (Figures 5(c) and 5(d)).

## 4. Discussion

In the present study, we tested the pluripotency and the expression of S1P1 of brain-derived NSCs. Moreover, these cells keep the ability of proliferation as we did before [18, 20]. Our data demonstrated that FTY720 could promote proliferation, differentiation of protoplasmic astrocytes, and migration of NSCs in a dose-dependent manner, because most of these effects were shown in higher concentration groups. Additionally, we found out that there were more matured oligodendrocytes on the FTY intervention groups.

Harada and his colleagues examined the effects of S1P on neural progenitor cells and reported that S1P induced proliferation of neural progenitor cells via S1P receptors (S1P1/3) activation and phosphorylation of extracellular signal-regulated kinase (ERK) by Brd-U incorporation assay [21]. This opinion was supported by another study about embryonic neural stem cells. However, other studies on lymphocytes and astrocytes have indicated the S1P-analog FTY720 plays a dual role in activation or inhibition on S1P1 [9, 22]. Therefore, the role of FTY720 on NSCs might not be the same with endogenous S1P.

Studies have shown that FTY720 increases the viability and neurogenic potential of irradiated NSCs of the hippocampus [23, 24]. For example, Stessin et al. reported more neuronal (NeuN positive) differentiation in the presence of 10 nM FTY720 in the irradiated brain, which counteracted the radiation-induced suppression of NSCs neurogenic potential. However, we failed to observe similar results in cultured NSCs, as no significant differences were found among groups of *β*-tubulin III positive cells. Our conclusion was supported by a study on a viral-induced demyelination mice model [16]. And no neuron differentiation on embryonic hippocampal NSCs has also been reported recently [25].

Surprisingly, the number of type-2 astrocytes, also known as the protoplasmic astrocyte, was much more in higher concentrations of FTY720, while the total amount of GFAP protein levels was not affected. Similar results have shown recently that the 3 major cell types differentiation were not affected by FTY using western blot [25]. Therefore, we speculate that FTY720 might be able to keep a relatively “stable differentiation model” of NSCs. Although coculture of protoplasmic astrocytes with NSCs promotes differentiation of neurons [15], S1PRs in protoplasmic astrocytes might be negatively regulated by FTY720 [9], resulting in suppression effects on neuronal differentiation, which might be another explanation why we did not observe neuronal preferences. However, as we know that protoplasmic astrocytes are mainly distributed in gray matter and participate in the formation of the blood-brain barrier, they are considered to be a benefit in CNS repair. Therefore, it would be one of the possible mechanisms of FTY720's neuroprotective effects in CNS disorders [26, 27]. Evidence about whether FTY have the ability to promote the development of oligodendrocytes remains controversial [28, 29]. Here, we found no induction of more oligodendrocyte lineage cells by FTY treated NSCs. However, more morphological matured oligodendrocytes were seen in the higher dose FTY720 groups. The different results among groups might be because of people using different originated NSCs or diseases models [1, 16, 29–31].

Migration ability is one of the premises of NSCs in repairing CNS injuries. In this study, FTY720 stimulates chemoattractant activity of NSCs from the upper chamber to the lower in Transwell assay. The migration activity seems to be strengthening with FTY720 concentration increase. This was further verified by protein level of filamentous actin (F-actin). Similar results were found in NSCs transplantation studies because inhibition of S1P1/3 abolishes the chemoattractant activity of S1P/S1P receptors [17, 32]. Although activation of S1P2 would reduce migration according to Kimura's research, no reports mentioned FTY720 could combine this receptor [21, 32]. As there are multiple targets downstream FTY720/S1PRs, it is not clear which signal pathway underlies the mechanism. Recently, Kassmer et al. reported that the migratory response of germline progenitor cells is dependent on activation of PI3K and Rac1 downstream of the S1P1 [33]. This G-protein-coupled receptors mediate cellular responses to attractive chemokines in a variety of cell types, including NSCs.

Some inadequacies need to be improved in further work. On the one hand, molecular pathways underlying the NSCs biological behavior in the presence of FTY720 were not tested. On the other hand,* in vivo* experiments need to be introduced to the present study. Though it is not certain whether FTY720's neuroprotective effects have correlations with NSCs, we believe that the application of this drug will be confined not only to MS but also to CNS injuries and other degenerative diseases.

## Supplementary Material

Nestin and S1P1 double positive confirmed the NSCs do express S1Ps. Note that the S1P1 was not as strong as Nestin.
