# Supplementary material for: Effects of FTY720 (Fingolimod) on Proliferation, Differentiation, and Migration of Brain-Derived Neural Stem Cells
Source: Stem Cells Int. 2016 Oct 18;2016:9671732. doi: 10.1155/2016/9671732 (PMC5088305; doi:10.1155/2016/9671732)
Supplement: Supplementary file 1 — Nestin and S1P1 double positive confirmed the NSCs do express S1Ps. Note that the S1P1 was not as strong as Nestin. [file 9671732.f1.pdf]

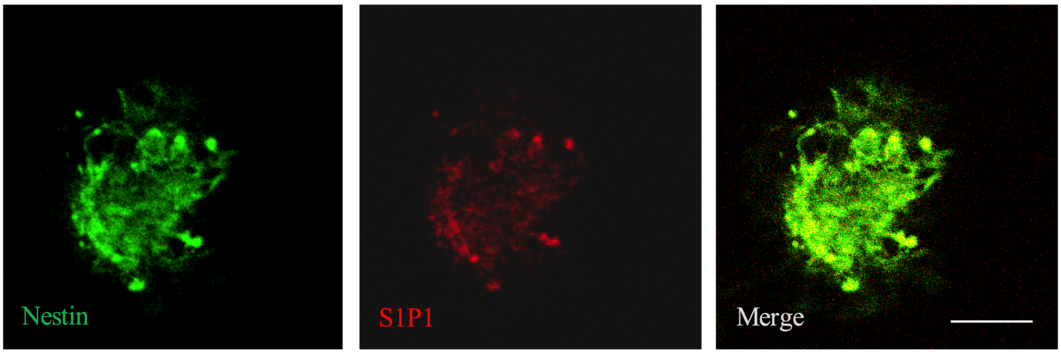

Supplement Figure 1 Images of immunostaining of NSCs with S1P1 and Nestin.

Nestin and S1P1 double positive confirmed the NSCs do express S1Ps. Note that the S1P1 is not as strong as Nestin. Bar = 80 μm.

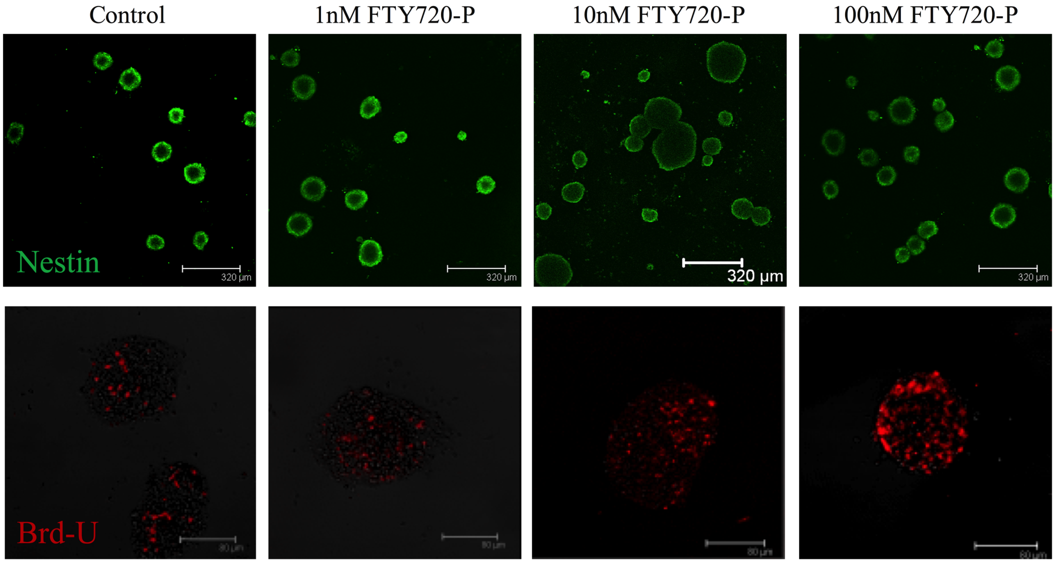

Supplement Figure 2 Immunocytochemistry staining for NSCs with Nestin (green) or Brd-U (red) in different groups. Note that the neurospheres were bigger in the 10 and 100 nM FTY720 groups and the number of Brd-U positive cells were much more than the control and lower concentration groups. Bar = 80 μm.
